# *Ligusticum chuanxiong* and *Paeonia lactiflora* **:** a review of their regulation of iron metabolism and ferroptosis in atherosclerosis therapy

**DOI:** 10.3389/fphar.2026.1831703

**Published:** 2026-07-15

**Authors:** Miao Zhang, WenBo Wei, ZongZheng Chen, Guipeng Xu, Qiting Chen, Xiaoshu Liu, FengQin Xu, ZhiXiu Lin

**Affiliations:** 1 School of Chinese Medicine, Faculty of Medicine, The Chinese University of Hong Kong, Hong Kong, Hong Kong SAR, China; 2 Shenzhen Second People’s Hospital, First Affiliated Hospital of Shenzhen University, Shenzhen, Guangdong, China; 3 Liaoning Vocational College of Medicine, Liaoning, China; 4 Department of Cardiovascular Disease, Beijing Xiyuan Hospital, China Academy of Chinese Medical Sciences, Beijing, China

**Keywords:** atherosclerosis, chishao, chuanxiong, ferroptosis, inflammation, iron metabolism, oxidative stress, traditional Chinese medicine

## Abstract

**Ethnopharmacological Relevance:**

Chuanxiong (*Ligusticum chuanxiong* Hort.) and Chishao (*Paeonia lactiflora* Pall.) are widely used in Traditional Chinese Medicine (TCM) for their ability to promote blood circulation and resolve blood stasis, particularly in the treatment of cardiovascular diseases such as atherosclerosis (AS).

**Aim of The Study:**

This review systematically summarizes the potential mechanisms by which *L. chuanxiong* and *P. lactiflora* modulate iron metabolism, inhibit ferroptosis, and enhance blood flow, offering innovative strategies for the treatment of AS.

**Materials and Methods:**

A comprehensive literature search was performed using databases such as PubMed, Web of Science, and CNKI. We implemented a narrative review with explicit search strategies across regional databases that included specific keywords related to the pharmacological effects of Chuanxiong and Chishao, particularly their roles in iron metabolism and ferroptosis. Studies were selected based on predefined inclusion criteria, ensuring relevance and quality. The data were organized to summarize the mechanisms of action and therapeutic potential of these substances, while also incorporating insights from traditional medical texts, including classical TCM literature. Specific search strategy: SU=((“Atherosclerosis” [MeSH] OR “Atherosclerotic plaque” [Title/Abstract] OR “AS” [Title/Abstract] OR “Arteriosclerosis” [Title/Abstract]) AND (“Chuanxiong” [Title/Abstract] OR “Ligusticum chuanxiong” [MeSH]) AND (“Chishao” [Title/Abstract] OR “Paeonia lactiflora” [MeSH])) AND ((“iron metabolism” [Title/Abstract] OR “ferroptosis” [MeSH] OR “lipid peroxidation” [Title/Abstract]) OR (“JAK-STAT pathway” OR “PI3K/AKT” OR “MAPK/NF-κB” OR “GPX4” [Title/Abstract]) AND (“randomized controlled trial” [Publication Type] OR “*in vitro*” [Title/Abstract] OR “*in vivo*” [Title/Abstract] OR “clinical study” [Title/Abstract])).

**Results:**

Ligusticum chuanxiong: and *P. lactiflora* modulate iron homeostasis by suppressing hepcidin and enhancing ferroportin, inhibit ferroptosis via GPX4 activation and System Xc^−^ upregulation, and mitigate oxidative stress/inflammation through ROS scavenging and NF-κB suppression, collectively reducing atherosclerotic plaque instability.

**Conclusion:**

Chuanxiong and Chishao demonstrate significant potential as therapeutic agents for AS by targeting iron metabolism and ferroptosis. Their traditional use in TCM is supported by modern pharmacological evidence, highlighting their potential for integration into modern therapeutic frameworks.

## 1 Introduction

Atherosclerosis (AS) is a multifactorial chronic inflammatory disease characterized by endothelial dysfunction, inflammation, oxidative stress, lipid accumulation, and plaque formation due to the buildup of lipids, immune cells, and fibrous metabolites in arterial walls (Libby, 2021; Falk, 2006). This condition significantly contributes to cardiovascular diseases, such as heart attacks and strokes. The progression of AS is influenced by various risk factors, including hyperlipidemia, hypertension, diabetes, and lifestyle choices like smoking and poor diet (Lechner et al., 2020). Despite advances in understanding AS, effective treatments remain limited, as traditional lipid-lowering therapies primarily focus on low-density lipoprotein (LDL) cholesterol while neglecting factors like oxidative stress, iron metabolism, and ferroptosis (Cheng et al., 2023). Emerging evidence suggests that iron overload and ferroptosis significantly contribute to the progression of AS, with ferroptosis—characterized by lipid peroxidation and iron accumulation—exacerbating endothelial injury and plaque instability (Fang et al., 2023). In Traditional Chinese Medicine (TCM), “blood stasis and obstruction” is the primary etiology and pathogenesis of AS and “promoting blood circulation and resolving stasis” is currently an effective strategy for preventing and treating the disease. Existing research indicates that “blood stasis and obstruction” is closely related to oxidative stress responses (Negre-Salvayre et al., 2020; Förstermann et al., 2017). TCM offers a unique perspective on cardiovascular health, leveraging botanical drugs such as the rhizome of Chuanxiong (*Ligusticum chuanxiong Hort.*) and the root of Chishao (*Paeonia lactiflora Pall.*) (Hao et al., 2017). These botanical drugs are traditionally prepared as decoctions or ethanol extracts and are clinically utilized for their ability to promote blood circulation and alleviate stasis (Peng et al., 2011; Rong XQ-q et al., 2023; Zheng et al., 2013), primarily attributed to bioactive constituents like Tetramethylpyrazine or ligustilide (Chuanxiong) and paeoniflorin (Chishao) (Table 1; Figure 2). Plant nomenclature follows The Plant List (www.Plantsoftheworldonline.org, *accessed 29.10.2025*) (Table 2; Table 3). This review (Figure 3) aims to elucidate their roles in regulating iron metabolism and suppressing ferroptosis, thereby exploring novel avenues for AS therapy.

**TABLE 1:** Extract preparation.

Parameter	*L. chuanxiong* Extract	*P. lactiflora* Extract
1. Solvent(s)	70% ethanol (v/v)	Distilled water
2. Extraction Method	Reflux (3 cycles, 1 h each)	Decoction (100 °C, 2 h)
3. Temperature/Duration	80 °C, 3 h total	100 °C, 2 h
4. Concentration	Rotary-evaporated, lyophilized (yield: 18.2% w/w)	Spray-dried (yield: 24.7% w/w)
5. Standardization	≥3.0% ferulic acid (HPLC-PDA)	≥6.0% paeoniflorin (HPLC-ELSD)
6. Storage	−20 °C in amber vials (stable ≥12 months)	4 °C with silica gel (stable ≥8 months)

**TABLE 2:** Plant material characterization.

Parameter	*Ligusticum chuanxiong* Hort. (Apiaceae)	*Paeonia lactiflora* Pall. (Paeoniaceae)
1. Botanical Identity	Binomial + authority: *Ligusticum chuanxiong* Hort	Binomial + authority: *Paeonia lactiflora* Pall
2. Plant Part Used	Rhizomes (dried)	Roots (peeled, dried)
3. Harvest Details	Sichuan, China (October 2022; 30.67°N, 103.92°E)	Anhui, China (September 2022; 31.83°N, 117.23°E)
4. Authentication	Voucher # CX202210-IMP (Deposited: KUN Herbarium)	Voucher # PL202209-IMP (Deposited: PE Herbarium)
5. Processing	Washed, sliced, shade-dried (35 °C), stored in dark (<25 °C)	Peeled, boiled 3 min, sun-dried, stored with desiccant

**TABLE 3:** Pharmacopeial drug names and standards.

Species	Region	Pharmacopeial drug name	Plant part	Key markers
*Conioselinum anthriscoides* cv. Chuanxiong	China	Chuanxiong Rhizoma (川芎)	Rhizome	Ligustilide (≥0.3%), Ferulic acid (≥0.02%)
​	Japan	Senkyu (センキュウ)	Rhizome	Senkyunolide A (≥0.2%)
​	Europe	Ligusticum Root	Root & Rhizome	Volatile oil (≥1.5% v/w)
*Paeonia lactiflora*	China	Chishao (赤芍)	Unpeeled root	Paeoniflorin (≥1.8%)
​	Korea	Jakhak (작약)	Peeled root	Albiflorin (≥0.5%)

## 2 Iron metabolism and ferroptosis in AS

Iron is an essential micronutrient that plays a critical role in various cellular functions, including oxygen transport, DNA synthesis, and electron transport in mitochondria. However, dysregulated iron homeostasis can have detrimental effects, particularly in the context of AS (Guo et al., 2023). Excess iron generates reactive oxygen species (ROS) through Fenton reactions, promoting oxidative stress and contributing to endothelial damage (Halliwell et al., 2023). Ferroptosis, a regulated form of cell death characterized by iron-dependent lipid peroxidation, has been implicated in the death of various cell types within atherosclerotic plaques, including endothelial cells, smooth muscle cells, and macrophages (Jiang et al., 2021; Xu X. et al., 2024). Notably, the impact of ferroptosis differs by cell type: ferroptosis in macrophages promotes necrotic core formation, whereas ferroptosis in vascular smooth muscle cells (VSMCs) contributes to plaque instability (Ouyang et al., 2021). The accumulation of lipid peroxides and the depletion of antioxidants such as glutathione lead to cellular dysfunction and death, exacerbating the progression of AS (Ouyang et al., 2021). Several key regulators are involved in the ferroptosis pathway, playing critical roles in maintaining cellular integrity and iron homeostasis. Glutathione Peroxidase 4 (GPX4) is a central enzyme that protects cells from oxidative damage by reducing lipid peroxides; its inhibition can trigger ferroptosis, underscoring its importance in cellular defense mechanisms (Bersuker et al., 2019). System Xc^−^, a cystine/glutamate antiporter, is essential for the uptake of cystine, which is required for glutathione synthesis. Dysregulation of System Xc^−^ leads to decreased glutathione levels, increasing susceptibility to ferroptosis (Wang et al., 2020). Additionally, iron regulatory proteins (IRPs) regulate iron homeostasis by controlling the expression of transferrin receptor 1 (TfR1) and ferritin (Silvestre et al., 2017). Alterations in IRP activity can result in iron overload, further promoting ferroptosis and exacerbating atherosclerotic lesion progression (Cardona and Montgomery, 2023). Dysregulation of these pathways collectively increases oxidative stress and ferroptosis susceptibility, accelerating the progression of AS (Kattoor et al., 2017).

The interplay between oxidative stress, iron overload, and ferroptosis creates a vicious cycle that amplifies inflammation and plaque instability (Wang et al., 2021). Elevated ROS levels contribute to endothelial dysfunction, inflammatory responses, and smooth muscle cell proliferation, while excess iron further fuels ROS generation and oxidative damage (Nakamura et al., 2019). Disruptions in iron regulatory mechanisms, such as abnormal hepcidin and ferroportin levels, exacerbate iron accumulation and inflammatory processes (Izumi et al., 2023). Targeting iron metabolism and ferroptosis pathways presents a promising therapeutic strategy for AS (Vinchi et al., 2020). Modulating GPX4 activity, enhancing System Xc^−^ function, and restoring IRP-mediated iron homeostasis could reduce oxidative stress, improve endothelial function, and slow disease progression (Fang et al., 2019). This emerging understanding underscores the critical role of iron dysregulation and ferroptosis in AS pathogenesis, offering novel avenues for therapeutic intervention.

## 3 Mechanisms of action of Chuanxiong and Chishao

Research indicates that Chuanxiong and Chishao have multifaceted effects on iron metabolism, ferroptosis, inflammation, and oxidative stress, positioning them as promising therapeutic candidates for AS (Gao et al., 2024).

### 3.1 Iron chelation and regulation of iron homeostasis

Chuanxiong and Chishao have been shown to chelate excess iron in the body, thereby reducing iron overload and subsequent production of ROS (Yuan et al., 2023a). These botanical drugs modulate the expression of IRPs, which control the balance of iron uptake, storage, and utilization. Additionally, they influence hepcidin, a key hormone that regulates systemic iron homeostasis (Wunderer et al., 2020). By downregulating hepcidin expression, these botanical drugs may enhance iron export from cells, reducing intracellular iron accumulation and its associated oxidative damage (Zhang et al., 2016). The active component TMP inhibits hepcidin transcription by suppressing the Stat3 and BMP6-SMAD1/5/8 signaling pathway, reduced hepcidin levels prevent FPN1 degradation, enhancing iron export from macrophages and vascular smooth muscle cells (Zhang et al., 2019; Wang Y. et al., 2025). Increasing *in vivo* and *in vitro* evidence supports this efficacy, but clinical support remains still lacking.

### 3.2 Inhibition of ferroptosis

Chuanxiong and Chishao enhance the activity of glutathione peroxidase 4 (GPX4), a critical enzyme that prevents lipid peroxidation and protects cells from ferroptosis (Yang and Stockwell, 2016; Lou et al., 2024). They also may upregulate the System Xc^−^ pathway, which is responsible for cystine uptake and glutathione synthesis. By increasing intracellular glutathione levels, these botanical drugs bolster cellular antioxidant defenses, mitigating ferroptosis (Dixon et al., 2012; Xu M. et al., 2024). Furthermore, they suppress the expression of pro-ferroptotic genes, such as acyl-CoA synthetase long-chain family member 4 (ACSL4) and TfR1, which are involved in lipid peroxidation and iron uptake, respectively (Doll et al., 2017; Zhang et al., 2025). The active component TMP orchestrates a concerted regulation of ferroptosis-related proteins, downregulating the iron-importer TFR1 while concurrently upregulating the key antioxidant regulator Nrf2 and its downstream target GPX4, thereby suppressing ferroptosis (Wang J. et al., 2025). The other active component PF can upregulate NRF2/SLC7A11/GPX4 to reduce ferroptosis, and directly suppressing HMGB1 to inhibit its-mediated ferroptosis (Chen et al., 2025; Wang et al., 2024). Given the distinct roles of ferroptosis in macrophages versus VSMCs within atherosclerotic plaques, understanding which cell types are preferentially targeted by these herbal interventions will be crucial for optimizing their therapeutic effects. Current evidence suggests that the above-mentioned herbal compounds may exert protective effects on both cell types, but further studies are needed to dissect cell-specific responses. Model consistency is generally good *in vitro*, however, further research is needed to evaluate these findings across different species and clinical models.

### 3.3 Anti-inflammatory effects

The bioactive metabolites in Chuanxiong and Chishao, such as ligustilide and paeoniflorin, have been shown to reduce the production of pro-inflammatory cytokines, including tumor necrosis factor-alpha (TNF-α) and interleukin-6 (IL-6) (Li X. et al., 2023). These botanical drugs also inhibit nuclear factor-kappa B (NF-κB) signaling, a central pathway in the regulation of inflammation. By attenuating NF-κB activation, they reduce the expression of inflammatory mediators, thereby alleviating chronic inflammation associated with AS (Xin et al., 2019; Wu et al., 2015). Both *in vitro* and *in vivo* experiments provide compelling evidence for their protective effects, yet there is a scarcity of human data to support these findings.

### 3.4 Antioxidant properties

Chuanxiong and Chishao exhibit potent antioxidant effects by scavenging ROS and enhancing the activity of endogenous antioxidant enzymes, such as superoxide dismutase (SOD) and catalase (Gao et al., 2024; Qin et al., 2022). These mechanisms help neutralize oxidative stress and protect cells from damage caused by excessive ROS production. The botanical drugs also reduce lipid peroxidation, a key driver of ferroptosis, further contributing to their cytoprotective effects (Zhang et al., 2021).

### 3.5 Role of TMP and PF in AS improvement

Tetramethylpyrazine (TMP), the active metabolite of Chuanxiong, and paeoniflorin (PF), the active metabolite of Chishao, have shown significant potential in improving AS by targeting iron overload and ferroptosis (Zhang et al., 2016; Zhang et al., 2019). These metabolites help restore iron homeostasis by reducing excess iron levels, which decreases the production of ROS and mitigates oxidative stress and inflammation in AS (Sun et al., 2018; Yuan et al., 2023b). TMP and PF exert protective effects on vascular cells, potentially preventing ferroptosis and preserving endothelial function, thereby enhancing vascular stability and reducing plaque vulnerability (Yuan et al., 2018; Wu et al., 2024; Zhu et al., 2024). Additionally, both TMP and PF possess antioxidant and anti-inflammatory properties, which further stabilize plaques and improve overall cardiovascular health by mitigating oxidative stress and inflammation (Zhang et al., 2022; Lu et al., 2024).

### 3.6 Synergistic effects

The combination of Chuanxiong and Chishao demonstrates synergistic effects in regulating iron metabolism, inhibiting ferroptosis, and providing anti-inflammatory and antioxidant benefits (Wang et al., 2017). Xiong-Shao Capsule is demonstrated to be safe and effective in reducing post-PCI recurrent angina and inhibiting luminal restenosis after PCI in senile CHD patients, compared to placebo, (Zheng et al., 2013; Shang et al., 2011). Evidence from clinical and animal studies indicates that Xuefu Zhuyu Decoction not only improves blood stasis syndrome but also overcomes aspirin resistance in patients with coronary heart disease (Li J. et al., 2023; Ma Q. et al., 2022; Xue et al., 2015). While both compounds contribute to ferroptosis suppression (Wang et al., 2017), TMP appears to exert a more dominant effect on restoring cellular iron export via the HEP/FPN axis (Zhang et al., 2016; Zhang et al., 2019), whereas PF shows a more prominent role in directly enhancing GPX4-mediated lipid peroxide scavenging and activating the Nrf2 antioxidant pathway (Wu et al., 2024). This synergy enhances their multi-target therapeutic potential, making them a promising approach for managing diseases characterized by iron dysregulation and oxidative stress, such as.

Collectively, these multi-target mechanisms demonstrate significant potential for mitigating iron dysregulation and ferroptosis in AS pathologies, bridging traditional herbal medicine with contemporary molecular therapeutics.

## 4 Discussion

### 4.1 Traditional use of Chuanxiong and Chishao in TCM

*Ligusticum chuanxiong*, known as “Chuanxiong” in Chinese, is renowned for its ability to promote blood circulation and resolve blood stasis, while *P. lactiflora*, known as “Chishao,” is valued for its blood-cooling and stasis-resolving properties (Yuan et al., 2019). The botanical drug pair of Chuanxiong and Chishao is derived from the classic TCM formula Xuefu Zhuyu Decoction, which has been used since the Qing Dynasty to treat blood stasis syndrome. This formula is particularly effective in alleviating chest pain, palpitations, and other symptoms associated with coronary heart disease (CHD) and AS (Yu et al., 2024; Yuan et al., 2017). Academician Chen Keji, a renowned TCM expert, frequently employs this botanical drug pair to treat patients with coronary heart disease, especially after stent implantation, highlighting its clinical significance in modern TCM practice (Yuerong et al., 2015).

In TCM, “Blood Stasis” reflects failed vascular circulation. Analogously, at the molecular level, iron overload can be viewed as a failure of cellular “iron flow” — a disruption of iron sequestration versus export. Central to this flow is the Hep/FPN axis: its dysregulation impairs iron export, leading to intracellular iron accumulation, lipid peroxidation, and ultimately ferroptosis (Fang et al., 2023; Wang J. et al., 2025; Chen et al., 2025; Wang et al., 2024). Hence, pathological iron buildup can be conceptualized as “Iron-Stasis” — cellular stagnation of iron flow parallel to vascular stasis. Restoring this flow via the Hep/FPN axis therefore represents a promising anti-atherosclerotic strategy. Notably, TMP and PF may exert such effects: emerging evidence shows TMP modulates hepcidin/ferroportin signaling to ameliorate iron disorders (Zhang et al., 2016; Zhang et al., 2019). Future studies on TMP/PF targeting this axis will help validate the “metabolic flow” theory and bridge TCM blood-stasis resolution with iron metabolism modulation.

**FIGURE 1:**
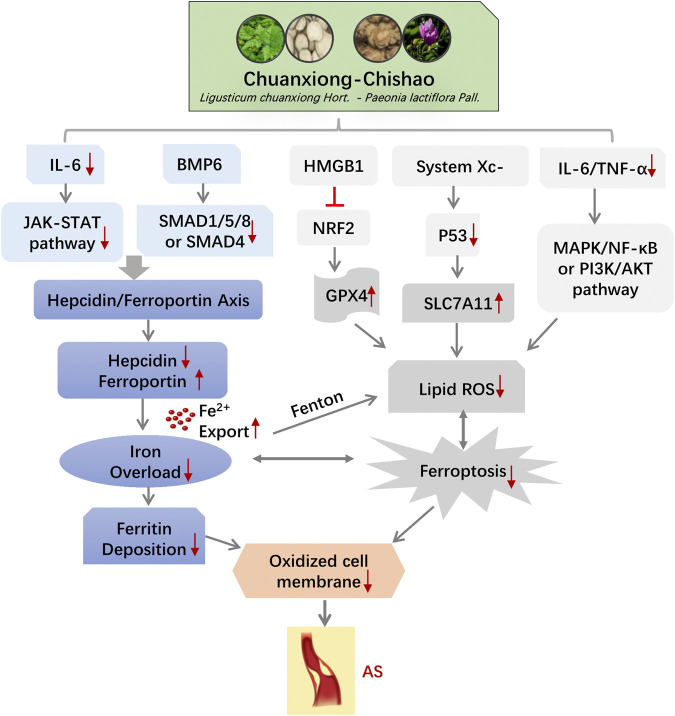
Graphical abstract schematic diagram of the mechanisms by which chuanxiong and chishao regulate iron metabolism and inhibit ferroptosis in AS.

The traditional use of Chuanxiong and Chishao is supported by modern pharmacological studies (Table 4; Table 5), which have identified their active metabolites, such as tetramethylpyrazine (TMP) from Chuanxiong and paeoniflorin (PF) from Chishao (Gao et al., 2024; Li et al., 2022). These metabolites are responsible for the botanical drugs ' cardiovascular benefits, including their ability to modulate iron metabolism, inhibit ferroptosis, and reduce oxidative stress and inflammation (Li X. et al., 2023; Yuan et al., 2017). This bridge between traditional use and modern scientific assessment underscores the potential of Chuanxiong and Chishao as innovative therapeutic agents for AS (Figure 1). The traditional use of these botanical drugs in treating blood stasis and cardiovascular diseases aligns with their modern pharmacological mechanisms (Zhang et al., 2024). For example, their ability to resolve blood stasis in TCM theory correlates with their modern roles in reducing oxidative stress, modulating iron metabolism, and inhibiting ferroptosis, all of which are critical in the pathogenesis of AS (Negre-Salvayre et al., 2020; Ma J. et al., 2022). Pharmacokinetic-pharmacodynamic (PK-PD) (Table 6)considerations indicate that, given the *in vitro* concentrations of TMP (100–300 µM) far exceed its clinically achievable plasma levels (≈9.4 µM) and that PF has negligible oral bioavailability, caution is needed when extrapolating current mechanistic data to clinical settings, and future PK-PD studies are warranted (Heo et al., 2016; Shen et al., 2013). This dual perspective not only investigates the traditional use of these botanical drugs but also provides a scientific basis for their application in modern medicine.

**TABLE 4:** Summary of pharmacological effects of *Ligusticum chuanxiong and Paeonia lactiflora* from published studies.

Model/Study system	Intervention	Duration	Outcomes/Measures	Main findings
FPN1 Tek-Cre Mice (Sun et al., 2018)	TMP (40 mg/kg/day)	7 days	Hepcidin expression, oxidative stress, inflammation factor	Hepcidin ↓ ROS↓, MDA↓, SOD↑ IL-1↓, IL-6↓
C57BL/6 mice (Zhang et al., 2019)	TMP (40 mg/kg/day)	7 days	Hepcidin expression, Serum iron	Hepcidin ↓, Serum iron ↓
ApoE^−/−^ mice (Xiao et al., 2021)	HFD + iron diet	16 weeks	Hepcidin expression, iron, oxidative stress	Hepcidin ↑, serum iron↑, liver iron↑, MDA↑, ferritin-H/L↑
DCM mice (Wu et al., 2024)	PF (20 mg/kg/day, 70 mg/kg/day)	8 weeks	Ferroptosis, oxidative stress, heart iron	GPX4↑ Fe^2+^ in heart↓ MDA↓
Ox-LDL-induced HUVECs (Yuan et al., 2023a)	TMP-PF (1 μmol/L TMP combined with 10 μmol/L PF)	24 h	angiogenesis in AS	NR4A1↓ cell proliferation↓, cell migration↓
Transgenic zebrafifish (Wang et al., 2017)	Chuanxiong-Chishao Herb pair extract	48 h	angiogenic activity	ESRα↑

**TABLE 5:** Known pharmacological functions for chuanxiong & chishao.

Herb	Main active metabolites	Chemical classification	Known pharmacological functions
Chuanxiong	Tetramethylpyrazine (TMP)	Alkaloid	Anti-platelet aggregation, cardiovascular protection, dilation of blood vessels, anti-inflammatory, antioxidant, anti-tumor, neuroprotection
	Ferulic Acid	Phenolic Acid	Inhibition of platelet aggregation, antioxidant, protection against myocardial and cerebral ischemia
	Ligustilide (e.g., Z-ligustilide)	Phthalide	Anti-inflammatory, smooth muscle relaxation (antispasmodic), potential anti-tumor activity
Chishao	Paeoniflorin	Monoterpene Glycoside	Anti-inflammatory, antioxidant, neuroprotective, antidepressant-like, cardioprotective, immunoregulatory, analgesic
	Paeoniflorin Metabolites (e.g., Benzoic acid derivatives)	-	The pharmacological effects of Paeoniflorin are believed to be largely generated by its metabolites, which may exert effects like antidepressant activity by crossing the blood-brain barrier
	Other compounds (Flavonoids, Tannins, etc.)	Various	The plant contains a wide profile of metabolites (over 1100 detected in flowers) that contribute to its overall antioxidant and anti-melanogenic activities

**FIGURE 2:**
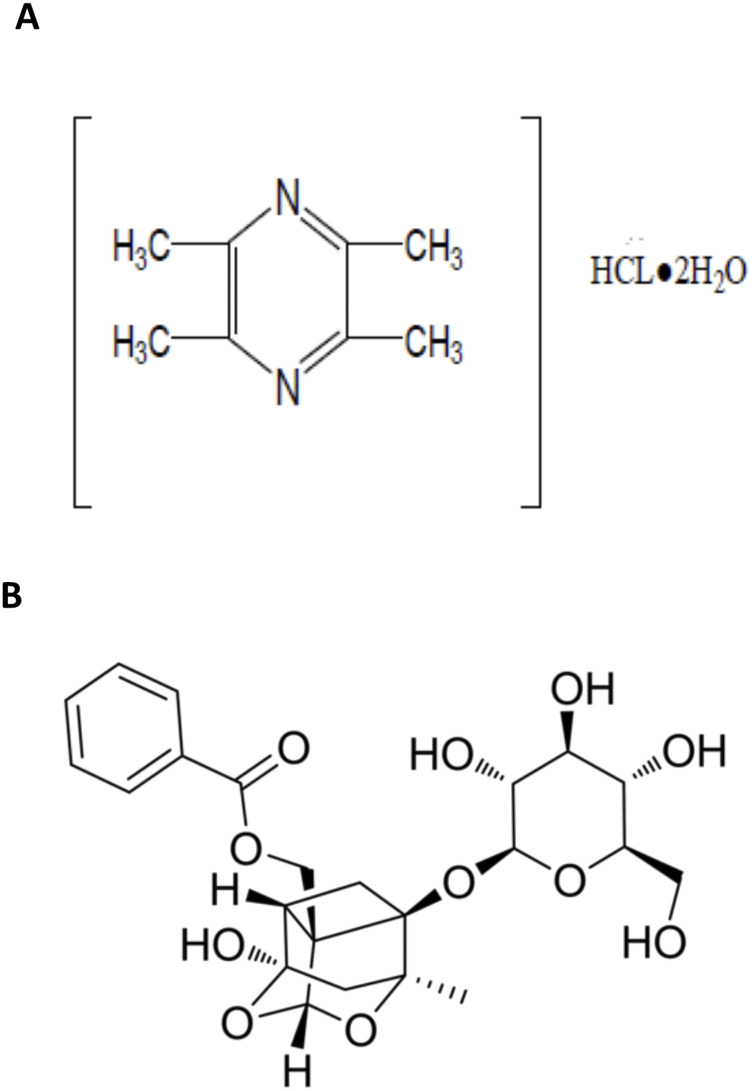
Chemical structures of tetramethylpyrazine (TMP), **(A)** the active component of Chuanxiong (*Ligusticum chuanxiong*), and paeoniflorin (PF), **(B)** the active component of Chishao (*Paeoniae Radix Rubra*).

**FIGURE 3:**
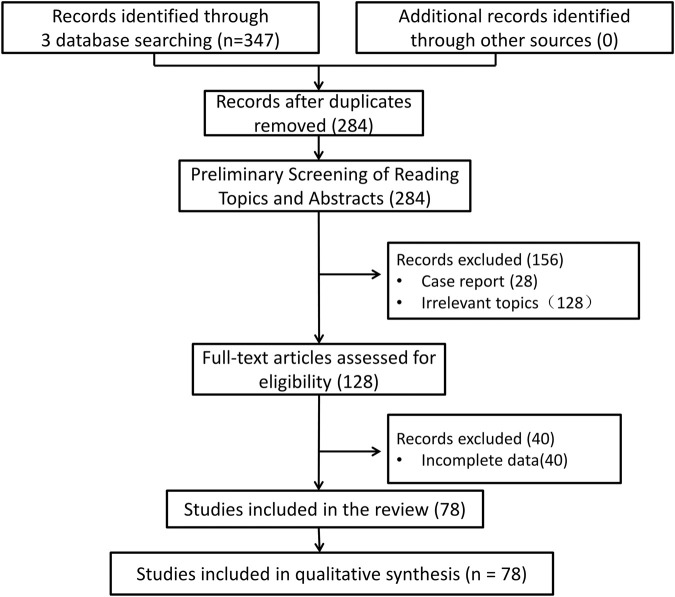
Flow diagram of study selection and identification.

**TABLE 6:** Pharmacological evidence for TMP & PF (core active components).

Active component (herb)	Pharmacological category	Key effects & quantitative data	Experimental model
Tetramethylpyrazine (TMP)*Ligusticum chuanxiong*	Pharmacokinetics	Plasma T_max_ of 15 min, C_max_ of 2866.43 ± 135.39 μg L^-1^, AUC_0-t_ of 241463.30 ± 28070.31 μg min·L^-1^, MRT_0-t_ of 353.13 ± 47.73 min, and CL_Z of 0.73 L min·kg^-1^, while anterior cingulate gyrus (ACC) dialysate shows T_max_ of 30 min, C_max_ of 1462.14 ± 197.38 μg L^-1^, AUC_0-t_ of 213115.62 ± 32570.07 μg min·L^-1^, and MRT_0-t_ of 172.16 ± 12.72 min	Rat model of spared sciatic nerve injury (Libby, 2021)
	Ferroptosis Inhibition	TMP inhibits transferrin receptor (TfR) expression by 35%–45% and intracellular reactive oxygen species (ROS) levels by 40%–50% in renal tubular epithelial cells during contrast-induced nephropathy, thereby attenuating ferroptosis with a 38%–48% reduction in lipid peroxide (LPO) and increasing cell viability to 65%–75%	Rats and HK-2 cells (Falk, 2006)
	Drug-Drug Interaction	TMP with aspirin and clopidogrel exerts a synergistic antithrombotic effect in rabbits, significantly reducing thrombus wet weight, platelet aggregation rate and serum platelet-activating factor (PAF) level compared with the single-drug groups, without obvious adverse drug interactions	Rabbits (Lechner et al., 2020)
Paeoniflorin (PF)*Paeonia lactiflora*	Pharmacokinetics	The terminal elimination half-life (t_1_/_2_) = 94.16 min; PF has an oral bioavailability of ∼3%–4% as a pure solution, while its glycyrrhizic acid micelle formulation increases C_max_ by ∼2.18-fold and AUC_0-t_ by ∼3.64-fold, significantly enhancing oral absorption	Rats (Cheng et al., 2023; Fang et al., 2023)
	Iron Metabolism Regulation and Ferroptosis Inhibition	Reshaped gut microbiota and promot its beneficial metabolites to enhance antioxidant capacity and upregulate GSH,GPX4 expression and restore iron metabolism homeostasis, maybe through Nrf2/HO-1 or PI3K/Akt signaling pathway, thereby inhibiting lipid peroxidation to confer ferroptosis resistance	DCM Mice (Negre-Salvayre et al., 2020)

### 4.2 Therapeutic implications and limitations

The dual role of Chuanxiong and Chishao in modulating iron metabolism and inhibiting ferroptosis positions them as promising therapeutic agents for AS (Chen et al., 2023; Zhi et al., 2023). Research indicates that these botanical drugs effectively reduce inflammation by modulating inflammatory pathways, thereby helping to decrease the progression of AS (Zhi et al., 2023; Liu et al., 2022). Clinical studies suggest that they enhance iron homeostasis by preventing the detrimental effects of iron overload, which is known to exacerbate cardiovascular conditions (Fang et al., 2023; Chen et al., 2020). Furthermore, research demonstrates that by inhibiting ferroptosis and protecting vascular cells from iron-dependent lipid peroxidation and cell death, the botanical drug pair may stabilize atherosclerotic plaques (Li et al., 2022; Yu et al., 2022). This stabilization is crucial, as unstable plaques are significant contributors to acute cardiovascular incidents such as heart attacks and strokes, thereby reducing the risk of cardiovascular events and offering a protective mechanism against acute complications (Kowara and Cudnoch-Jedrzejewska, 2021; Bułdak, 2022).

In summary, the integration of Chuanxiong and Chishao into treatment protocols could provide a multifaceted approach to managing AS, warranting further investigation and assessment in clinical settings (Libby et al., 2016). While Chuanxiong and Chishao show promise in treating AS, several limitations remain. Current evidence lacks positive controls in basic research and sufficient clinical trials, and safety evaluations (e.g., hepatotoxicity, drug interactions) are needed. Moreover, most mechanistic studies rely on the ApoE^−/−^ mouse model, which develops more severe iron overload and oxidative stress than human plaques, potentially over-representing the role of iron and inflating the apparent efficacy of herbal compounds. Therefore, findings from these models should be translated cautiously, and future studies using human tissue samples, alternative animal models, or clinical PK-PD correlations are urgently needed to validate the therapeutic potential of TMP and PF in AS.

### 4.3 Future directions

A critical but often overlooked issue in herbal research is potential herb-drug interactions, especially because AS patients are almost always on statins or antiplatelet agents (Guo et al., 2016). Regarding antiplatelet therapy, studies in rats have shown that co-administration of TMP with aspirin and clopidogrel dual antiplatelet therapy (DAPT) significantly altered pharmacokinetics: TMP increased the AUC of aspirin approximately six-fold while reducing the AUC of clopidogrel and the Cmax of its active metabolite by about 75%, and also prolonged prothrombin time (Wang et al., 2022). Regarding statin interactions, *in vitro* evidence indicates that peony (Paeonia lactiflora, the botanical source of PF) may inhibit CYP3A4, the primary metabolizing enzyme of many statins, theoretically increasing their plasma levels and clinical effects (Wang et al., 2015). Further research is urgently needed to systematically evaluate the safety and potential dose-sparing benefits of TMP and PF when co-administered with standard AS medications.

To translate the promising findings on Chuanxiong and Chishao into clinical practice, future research must address three critical pathways: first, elucidating their precise mechanisms of action in modulating iron metabolism and inhibiting ferroptosis; second, validating their efficacy and safety through rigorous randomized controlled trials in diverse patient cohorts; and ultimately, exploring their synergistic potential when integrated with conventional therapies like statins or antiplatelet agents. Addressing these priorities is essential for establishing this herbal pair as an evidence-based, complementary strategy within mainstream cardiovascular therapeutics.

## 5 Conclusion

Preliminary evidence suggests *that* Chuanxiong and Chishao **may offer potential** innovative strategies for the treatment and management of AS by targeting iron metabolism and ferroptosis. Their multifaceted mechanisms of action—including iron chelation, antioxidant activity, and anti-inflammatory effects—indicate their possible utility as adjunctive or alternative therapies (Figure 1). While preclinical studies show promise, rigorous clinical assessment is essential to confirm their efficacy and safety in humans. Further studies must address existing knowledge gaps—such as mechanistic clarity, clinical efficacy, and integration with conventional therapies—before these metabolites can be integrated into mainstream practice*.* Ultimately, with robust evidence, Chuanxiong and Chishao **could** pave the way for novel approaches to AS management, bridging traditional medicine and contemporary biomedical science.

## Glossary

ACSL4: Acyl-CoA Synthetase Long-Chain Family Member 4; a key enzyme in ferroptosis that catalyzes the activation of long-chain fatty acids (e.g., arachidonic acid) into acyl-CoA esters, promoting lipid peroxidation
Atherosclerosis (AS): A chronic inflammatory vascular disease characterized by the accumulation of lipids, immune cells, and extracellular matrix in arterial walls, leading to plaque formation and vascular stenosis
Signal Transducer and Activator of Transcription 3 (Stat3): A member of the Stat family of transcription factors; activated by cytokines (e.g., IL-6) or growth factors via tyrosine phosphorylation, translocates to the nucleus, and regulates the transcription of target genes (e.g., hepcidin in iron metabolism). In atherosclerosis, aberrant Stat3 activation promotes hepcidin overexpression, leading to FPN1 degradation and iron overload in plaques
BMP6-SMAD1/5/8 Signaling Pathway: A key pathway regulating systemic iron homeostasis; Bone Morphogenetic Protein 6 (BMP6) (secreted by hepatocytes and macrophages) binds to its cell surface receptors, triggering the phosphorylation and activation of SMAD1/5/8. Activated SMAD1/5/8 forms a complex with SMAD4, translocates to the nucleus, and induces the transcription of hepcidin. In the context of Chuanxiong’s action, tetramethylpyrazine (TMP) inhibits this pathway to downregulate hepcidin, thereby preserving FPN1 and enhancing iron export
Ferroptosis: An iron-dependent form of regulated cell death driven by excessive lipid peroxidation, characterized by the depletion of GSH, inactivation of GPX4, and accumulation of lipid peroxides
**Cystine/Glutamate Antiporter (System Xc** ^**−**^ **)**: A plasma membrane transporter composed of SLC7A11 (light chain) and SLC3A2 (heavy chain); mediates cystine import and glutamate export, providing cystine for glutathione (GSH) synthesis
Ferritin Heavy Chain (FTH1): A subunit of ferritin (the major iron storage protein) with ferroxidase activity; regulates intracellular iron storage by oxidizing Fe^2+^ to Fe^3+^ for incorporation into ferritin
Ferroportin 1 (FPN1): The only known mammalian iron exporter protein, expressed on the surface of macrophages, hepatocytes, and enterocytes; mediates iron release into the circulation, with activity inhibited by hepcidin
Glutathione Peroxidase 4 (GPX4): A selenoprotein and key suppressor of ferroptosis; catalyzes the reduction of lipid peroxides (LPO) to non-toxic alcohols using glutathione (GSH) as a cofactor
Glutathione: A tripeptide (γ-glutamyl-cysteinyl-glycine) with antioxidant properties; serves as a cofactor for GPX4 to scavenge lipid peroxides and maintain redox homeostasis, preventing ferroptosis
HMGB1/NRF2/GPX4 Pathway: A multi-component signaling axis that regulates ferroptosis and oxidative stress responses in cells, involving High Mobility Group Box 1 (HMGB1), Nuclear Factor Erythroid 2-Related Factor 2 (NRF2), and Glutathione Peroxidase 4 (GPX4). Under physiological conditions, NRF2 activates the transcription of GPX4 and SLC7A11 (a cystine transporter), promoting glutathione (GSH) synthesis and GPX4-mediated lipid peroxide scavenging to suppress ferroptosis. When cells are under stress, HMGB1 is released from the nucleus and acts as a pro-ferroptotic factor—disrupting iron homeostasis and inhibiting NRF2 activity
Hepcidin: A peptide hormone primarily produced by hepatocytes; regulates systemic iron homeostasis by binding to FPN1, inducing its internalization and degradation, thereby reducing iron export
Lipid Peroxide: A product of lipid oxidation; accumulates during ferroptosis due to the failure of antioxidant systems (e.g., GPX4, GSH), leading to membrane damage and cell death
p53-SLC7A11 Pathway: A signaling pathway regulating ferroptosis; phosphorylated p53 (at Ser15) represses the transcription of SLC7A11 (a component of System Xc^−^), reducing cystine import and GSH synthesis, thereby inducing ferroptosis in pathological cells (e.g., foam cells)
Reactive Oxygen Species (ROS): Highly reactive molecules (e.g., superoxide, hydrogen peroxide) generated during cellular metabolism; excessive ROS production promotes lipid peroxidation and ferroptosis in atherosclerotic vascular cells
Superoxide Dismutase (SOD): An antioxidant enzyme that catalyzes the dismutation of superoxide radicals (O_2_^−^) into hydrogen peroxide (H_2_O_2_) and oxygen (O_2_); plays a role in mitigating oxidative stress and suppressing ferroptosis
Transferrin Receptor 1 (TfR1): A transmembrane protein that mediates the uptake of transferrin-bound iron (TBI) into cells; its expression is upregulated under iron deficiency via IRP2-IRE binding, promoting iron import
Traditional Chinese Medicine (TCM): A holistic medical system originating in China, emphasizing the balance of “Qi” (vital energy) and “Blood”; uses herbs (e.g., Chuanxiong, Chishao) and compound preparations to treat diseases like atherosclerosis, based on theories such as “activating blood and resolving stasis.”
Tetramethylpyrazine (TMP): A major active alkaloid isolated from *Ligusticum chuanxiong* (Chuanxiong); regulates iron metabolism (e.g., inhibits hepcidin) and suppresses ferroptosis (e.g., upregulates GPX4) in atherosclerosis
Paeoniflorin (PF): A primary monoterpene glycoside from *Paeonia lactiflora* (Chishao); modulates iron homeostasis (e.g., stabilizes IRP2) and inhibits ferroptosis (e.g., activates Nrf2-GCL-GSH axis) in atherosclerotic models
Xiong-Shao Capsule: A classic oral compound preparation in traditional Chinese medicine (TCM), composed of two core medicinal herbs—Ligusticum chuanxiong Hort. (Chuanxiong) and Paeonia lactiflora Pall. (Chishao)—at a weight ratio of 1:1. Guided by the TCM theory of “activating blood and resolving stasis,” it is clinically used for cardiovascular diseases such as stable coronary artery disease (CAD) and atherosclerosis
Xuefu Zhuyu Decoction: A classic TCM decoction (oral liquid preparation) with a multi-herb formula, taking Ligusticum chuanxiong Hort. (Chuanxiong) and Paeonia lactiflora Pall. (Chishao) as core components (supplemented by herbs like Angelica sinensis, Lycopus lucidus, etc.). Based on the TCM theory of “dispelling blood stasis and unblocking collaterals,” it targets blood stasis-related conditions, including atherosclerosis

## References

[B1] Bersuker K. Hendricks J. M. Li Z. Magtanong L. Ford B. Tang P. H. (2019). The CoQ oxidoreductase FSP1 acts parallel to GPX4 to inhibit ferroptosis. Nature 575 (7784), 688–692. 10.1038/s41586-019-1705-2 31634900 PMC6883167

[B2] Bułdak Ł. (2022). Cardiovascular Diseases-A focus on atherosclerosis, its prophylaxis, complications and recent advancements in therapies. Int. Journal Molecular Sciences 23 (9), 4695. 10.3390/ijms23094695 PMC9103939 35563086

[B3] Cardona C. J. Montgomery M. R. (2023). Iron regulatory proteins: players or pawns in ferroptosis and cancer? Front. Molecular Biosciences 10, 1229710. 10.3389/fmolb.2023.1229710 PMC10340119 37457833

[B4] Chen Y. Lu W. Yang K. Duan X. Li M. Chen X. (2020). Tetramethylpyrazine: a promising drug for the treatment of pulmonary hypertension. Br. Journal Pharmacology 177 (12), 2743–2764. 10.1111/bph.15000 31976548 PMC7236078

[B5] Chen Y. Li X. Wang S. Miao R. Zhong J. (2023). Targeting iron metabolism and ferroptosis as novel therapeutic approaches in cardiovascular diseases. Nutrients 15 (3), 591. 10.3390/nu15030591 36771298 PMC9921472

[B6] Chen S. Zheng W. Wang Y. Zhao X. Deng W. Chai N. (2025). Paeoniflorin attenuates cisplatin induced ototoxicity by inhibiting ferroptosis mediated by HMGB1/NRF2/GPX4 pathway. Food Chemical Toxicology An International Journal Published Br. Industrial Biol. Res. Assoc. 202, 115550. 10.1016/j.fct.2025.115550 40374000

[B7] Cheng J. Huang H. Chen Y. Wu R. (2023). Nanomedicine for diagnosis and treatment of atherosclerosis. Adv. Science Weinheim, Baden-Wurttemberg, Ger. 10 (36), e2304294. 10.1002/advs.202304294 PMC10754137 37897322

[B8] Dixon S. J. Lemberg K. M. Lamprecht M. R. Skouta R. Zaitsev E. M. Gleason C. E. (2012). Ferroptosis: an iron-dependent form of nonapoptotic cell death. Cell 149 (5), 1060–1072. 10.1016/j.cell.2012.03.042 22632970 PMC3367386

[B9] Doll S. Proneth B. Tyurina Y. Y. Panzilius E. Kobayashi S. Ingold I. (2017). ACSL4 dictates ferroptosis sensitivity by shaping cellular lipid composition. Nat. Chemical Biology 13 (1), 91–98. 10.1038/nchembio.2239 27842070 PMC5610546

[B10] Falk E. (2006). Pathogenesis of atherosclerosis. J. Am. Coll. Cardiol. 47 (8 Suppl. l), C7–C12. 10.1016/j.jacc.2005.09.068 16631513

[B11] Fang X. Wang H. Han D. Xie E. Yang X. Wei J. (2019). Ferroptosis as a target for protection against cardiomyopathy. Proc. Natl. Acad. Sci. U. S. A. 116 (7), 2672–2680. 10.1073/pnas.1821022116 30692261 PMC6377499

[B12] Fang X. Ardehali H. Min J. Wang F. (2023). The molecular and metabolic landscape of iron and ferroptosis in cardiovascular disease. Nat. Reviews Cardiol. 20 (1), 7–23. 10.1038/s41569-022-00735-4 PMC9252571 35788564

[B13] Förstermann U. Xia N. Li H. (2017). Roles of vascular oxidative stress and nitric oxide in the pathogenesis of atherosclerosis. Circulation Research 120 (4), 713–735. 10.1161/CIRCRESAHA.116.309326 28209797

[B14] Gao J. Wang N. Song W. Yuan Y. Teng Y. Liu Z. (2024). Mechanisms underlying the synergistic effects of chuanxiong combined with chishao on treating acute lung injury based on network pharmacology and molecular docking combined with preclinical evaluation. J. Ethnopharmacology 325, 117862. 10.1016/j.jep.2024.117862 38342157

[B15] Guo M. Liu Y. Shi D. (2016). Cardiovascular actions and therapeutic potential of tetramethylpyrazine (active component isolated from rhizoma chuanxiong): roles and mechanisms. Biomed. Res. Int. 2016, 2430329. 10.1155/2016/2430329 27314011 PMC4893570

[B16] Guo Q. Qian C. Qian Z. M. (2023). Iron metabolism and atherosclerosis. Trends Endocrinology Metabolism TEM 34 (7), 404–413. 10.1016/j.tem.2023.04.003 37210298

[B17] Halliwell B. Watt F. Minqin R. (2023). Iron and atherosclerosis: lessons learned from rabbits relevant to human disease. Free Radical Biology and Medicine 209 (Pt 1), 165–170. 10.1016/j.freeradbiomed.2023.10.383 37852545

[B18] Hao P. Jiang F. Cheng J. Ma L. Zhang Y. Zhao Y. (2017). Traditional Chinese medicine for cardiovascular disease: evidence and potential mechanisms. J. Am. Coll. Cardiol. 69 (24), 2952–2966. 10.1016/j.jacc.2017.04.041 28619197

[B19] Heo S. H. Lee D. S. Ham S. H. Cho J. H. Kwon Y. D. Lee Y. B. (2016). Pharmacokinetic evaluation of paeoniflorin after oral administration of paeoniae radix extract powder to healthy Korean subjects using UPLC-MS/MS. J. Pharm. Investig. 46, 273–282. 10.1007/s40005-016-0242-3

[B20] Izumi Y. Kataoka H. Koshiba A. Ito F. Tanaka Y. Takaoka O. (2023). Hepcidin as a key regulator of iron homeostasis triggers inflammatory features in the normal endometrium. Free Radical Biology and Medicine 209 (Pt 2), 191–201. 10.1016/j.freeradbiomed.2023.10.402 37884101

[B21] Jiang X. Stockwell B. R. Conrad M. (2021). Ferroptosis: mechanisms, biology and role in disease. Nat. Reviews Mol. Cell Biology 22 (4), 266–282. 10.1038/s41580-020-00324-8 PMC8142022 33495651

[B22] Kattoor A. J. Pothineni N. V. K. Palagiri D. Mehta J. L. (2017). Oxidative stress in atherosclerosis. Curr. Atherosclerosis Reports 19 (11), 42. 10.1007/s11883-017-0678-6 28921056

[B23] Kowara M. Cudnoch-Jedrzejewska A. (2021). Different approaches in therapy aiming to stabilize an unstable atherosclerotic plaque. Int. Journal Molecular Sciences 22 (9), 4354. 10.3390/ijms22094354 33919446 PMC8122261

[B24] Lechner K. von Schacky C. McKenzie A. L. Worm N. Nixdorff U. Lechner B. (2020). Lifestyle factors and high-risk atherosclerosis: pathways and mechanisms beyond traditional risk factors. Eur. Journal Preventive Cardiology 27 (4), 394–406. 10.1177/2047487319869400 31408370 PMC7065445

[B25] Li S. Liu P. Feng X. Wang Y. Du M. Wang J. (2022). The role and mechanism of tetramethylpyrazine for atherosclerosis in animal models: a systematic review and meta-analysis. PloS One 17 (5), e0267968. 10.1371/journal.pone.0267968 35500001 PMC9060352

[B26] Li X. Sun C. Zhang J. Hu L. Yu Z. Zhang X. (2023a). Protective effects of paeoniflorin on cardiovascular diseases: a pharmacological and mechanistic overview. Front. Pharmacology 14, 1122969. 10.3389/fphar.2023.1122969 PMC10267833 37324475

[B27] Li J. Guo Z. H. Liu J. H. Zhong S. J. Kuang H. F. Yang Y. (2023b). Efficacy and mechanism of xuefu zhuyu decoction on model rats of coronary heart disease with heart blood stasis syndrome based on metabolomics. Zhongguo Zhong Yao Za Zhi = Zhongguo Zhongyao Zazhi = China Journal Chin. Materia Medica 48 (20), 5623–5631. 10.19540/j.cnki.cjcmm.20230710.702 38114155

[B28] Libby P. (2021). The changing landscape of atherosclerosis. Nature 592 (7855), 524–533. 10.1038/s41586-021-03392-8 33883728

[B29] Libby P. Bornfeldt K. E. Tall A. R. (2016). Atherosclerosis: successes, surprises, and future challenges. Circulation Research 118 (4), 531–534. 10.1161/CIRCRESAHA.116.308334 26892955 PMC4762065

[B30] Liu H. Zhu L. Chen L. Li L. (2022). Therapeutic potential of traditional Chinese medicine in atherosclerosis: a review. Phytotherapy Research PTR 36 (11), 4080–4100. 10.1002/ptr.7590 36029188

[B31] Lou T. Wu H. Feng M. Liu L. Yang X. Pan M. (2024). Integration of metabolomics and transcriptomics reveals that Da Chuanxiong Formula improves vascular cognitive impairment via ACSL4/GPX4 mediated ferroptosis. J. Ethnopharmacology 325, 117868. 10.1016/j.jep.2024.117868 38325668

[B32] Lu Y. Yin L. Yang W. Wu Z. Niu J. (2024). Antioxidant effects of paeoniflorin and relevant molecular mechanisms as related to a variety of diseases: a review. Biomed. and Pharmacotherapy = Biomedecine and Pharmacotherapie 176, 116772. 10.1016/j.biopha.2024.116772 38810407

[B33] Ma Q. Cai Z. Sui L. Wang X. (2022a). Efficacy and safety of acupuncture combined with xuefu zhuyu decoction on major adverse cardiovascular events after percutaneous coronary intervention: a protocol for systematic review and meta-analysis. Medicine 101 (46), e31735. 10.1097/md.0000000000031735 36401381 PMC9678503

[B34] Ma J. Zhang H. Chen Y. Liu X. Tian J. Shen W. (2022b). The role of macrophage iron overload and ferroptosis in atherosclerosis. Biomolecules 12 (11), 1702. 10.3390/biom12111702 36421722 PMC9688033

[B35] Nakamura T. Naguro I. Ichijo H. (2019). Iron homeostasis and iron-regulated ROS in cell death, senescence and human diseases. Biochimica biophysica acta General Subj. 1863 (9), 1398–1409. 10.1016/j.bbagen.2019.06.010 31229492

[B36] Negre-Salvayre A. Guerby P. Gayral S. Laffargue M. Salvayre R. (2020). Role of reactive oxygen species in atherosclerosis: lessons from murine genetic models. Free Radical Biology and Medicine 149, 8–22. 10.1016/j.freeradbiomed.2019.10.011 31669759

[B37] Ouyang S. You J. Zhi C. Li P. Lin X. Tan X. (2021). Ferroptosis: the potential value target in atherosclerosis. Cell Death and Disease 12 (8), 782. 10.1038/s41419-021-04054-3 34376636 PMC8355346

[B38] Peng W. Shi D. Z. Xue Y. T. (2011). Effect of xiongshao capsule in treating 112 patients with coronary heart disease angina pectoris of xin-blood stasis syndrome. Zhongguo Zhong Xi Yi Jie He Za Zhi Zhongguo Zhongxiyi Jiehe Zazhi = Chin. Journal Integrated Traditional West. Medicine 31 (2), 191–194. 10.88888/j.1003-5370.2011.2.191-194 21425572

[B39] Qin Y. Chen F. Tang Z. Ren H. Wang Q. Shen N. (2022). Ligusticum chuanxiong hort as a medicinal and edible plant foods: Antioxidant, anti-aging and neuroprotective properties in *Caenorhabditis elegans* . Front. Pharmacology 13, 1049890. 10.3389/fphar.2022.1049890 PMC9643709 36386171

[B41] Rong Y. Qiqi X. Pengqi L. Yu M. Wei-hong C. (2023). Effect of chuanxiong rhizoma-paeoniae Radix rubra herb pair on differential regulation of angiogenesis in coronary heart disease world. J. Integr. Traditional West. Med. 18 (1), 209–212. 10.13935/j.cnki.sjzx.230138

[B42] Shang Q. H. Xu H. Lu X. Y. Wen C. Shi D. Z. Chen K. J. (2011). A multi-center randomized double-blind placebo-controlled trial of xiongshao capsule in preventing restenosis after percutaneous coronary intervention: a subgroup analysis of senile patients. Chin. Journal Integrative Medicine 17 (9), 669–674. 10.1007/s11655-011-0843-7 21910067

[B43] Shen T. Xu H. Weng W. Zhang J. (2013). Single- and multiple-dose pharmacokinetics of a novel tetramethylpyrazine reservoir-type transdermal patch versus tetramethylpyrazine phosphate oral tablets in healthy normal volunteers, and *in vitro*/In vivo correlation. Biol. Pharm. Bull. 36 (6), 931–937. 10.1248/bpb.b12-00909 23514701

[B44] Silvestre O. M. Gonçalves A. Nadruz W. Jr. Claggett B. Couper D. Eckfeldt J. H. (2017). Ferritin levels and risk of heart failure-the atherosclerosis risk in communities study. Eur. Journal Heart Failure 19 (3), 340–347. 10.1002/ejhf.701 27976478 PMC5334451

[B45] Sun M. Y. Zhang M. Chen S. L. Zhang S. P. Guo C. Y. Wang J. S. (2018). The influence of hyperlipidemia on endothelial function of FPN1 tek-cre mice and the intervention effect of tetramethylpyrazine. Cell. Physiology Biochemistry International Journal Experimental Cellular Physiology, Biochemistry, Pharmacology 47 (1), 119–128. 10.1159/000489754 29763925

[B46] Vinchi F. Porto G. Simmelbauer A. Altamura S. Passos S. T. Garbowski M. (2020). Atherosclerosis is aggravated by iron overload and ameliorated by dietary and pharmacological iron restriction. Eur. Heart Journal 41 (28), 2681–2695. 10.1093/eurheartj/ehz112 30903157

[B47] Wang W. Tian D. D. Zheng B. Wang D. Tan Q. R. Wang C. Y. (2015). Peony-glycyrrhiza decoction, an herbal preparation, inhibits clozapine metabolism via cytochrome P450s, but not flavin-containing monooxygenase in *in* Vitro models. Drug Metab. Dispos. 43 (7), 1147–1153. 10.1124/dmd.114.062653 25948710

[B48] Wang Y. Guo G. Yang B. R. Xin Q. Q. Liao Q. W. Lee S. M. (2017). Synergistic effects of chuanxiong-chishao herb-pair on promoting angiogenesis at network pharmacological and pharmacodynamic levels. Chin. Journal Integrative Medicine 23 (9), 654–662. 10.1007/s11655-017-2408-x 28551771

[B49] Wang L. Liu Y. Du T. Yang H. Lei L. Guo M. (2020). ATF3 promotes erastin-induced ferroptosis by suppressing system Xc. Cell Death Differentiation 27 (2), 662–675. 10.1038/s41418-019-0380-z 31273299 PMC7206049

[B50] Wang Y. Zhao Y. Ye T. Yang L. Shen Y. Li H. (2021). Ferroptosis signaling and regulators in atherosclerosis. Front. Cell Developmental Biology 9, 809457. 10.3389/fcell.2021.809457 PMC8716792 34977044

[B51] Wang K. Hao Y. Wang C. Zhao X. He X. Sun C. C. (2022). Simultaneous improvement of physical stability, dissolution, bioavailability, and antithrombus efficacy of aspirin and ligustrazine through cocrystallization. Int. J. Pharm. 25 (616), 121541. 10.1016/j.ijpharm.2022.121541 35124115

[B52] Wang Y. Hao Y. Yuan L. Tian H. Sun X. Zhang Y. (2024). Ferroptosis: a new mechanism of traditional Chinese medicine for treating ulcerative colitis. Front. Pharmacology 15, 1379058. 10.3389/fphar.2024.1379058 PMC11184165 38895617

[B53] Wang Y. Wu L. Wang H. Jiang M. Chen Y. Zheng X. (2025a). Ligusticum chuanxiong: a chemical, pharmacological and clinical review. Front. Pharmacology 16, 1523176. 10.3389/fphar.2025.1523176 PMC11996930 40235541

[B54] Wang J. Chen W. Wang F. Yang Q. Gao B. Zhang Y. (2025b). Tetramethylpyrazine ameliorates sepsis-induced liver injury via inhibiting ferroptosis. Biochem. Pharmacology 241, 117193. 10.1016/j.bcp.2025.117193 40721008

[B55] Wu Z. Uchi H. Morino-Koga S. Shi W. Furue M. (2015). Z-ligustilide ameliorated ultraviolet B-induced oxidative stress and inflammatory cytokine production in human keratinocytes through upregulation of Nrf2/HO-1 and suppression of NF-κB pathway. Exp. Dermatology 24 (9), 703–708. 10.1111/exd.12758 25977183

[B56] Wu H. Zhang P. Zhou J. Hu S. Hao J. Zhong Z. (2024). Paeoniflorin confers ferroptosis resistance by regulating the gut microbiota and its metabolites in diabetic cardiomyopathy. Am. Journal Physiology Cell Physiology 326 (3), C724–c741. 10.1152/ajpcell.00565.2023 38223927

[B57] Wunderer F. Traeger L. Sigurslid H. H. Meybohm P. Bloch D. B. Malhotra R. (2020). The role of hepcidin and iron homeostasis in atherosclerosis. Pharmacol. Research 153, 104664. 10.1016/j.phrs.2020.104664 31991168 PMC7066581

[B79] Xiao L. Luo G. Li H. Yao P. Tang Y.Y (2021). Dietary iron overload mitigates atherosclerosis in high-fat diet-fed apolipoprotein E knockout mice: role of dysregulated hepatic fatty acid metabolism. Biochim Biophys Acta Mol. Cell Biol. Lipids. 1866 (10), 159004. 10.1016/j.bbalip.2021.159004 34245925

[B58] Xin Q. Yuan R. Shi W. Zhu Z. Wang Y. Cong W. (2019). A review for the anti-inflammatory effects of paeoniflorin in inflammatory disorders. Life Sciences 237, 116925. 10.1016/j.lfs.2019.116925 31610201

[B59] Xu X. Xu X. D. Ma M. Q. Liang Y. Cai Y. B. Zhu Z. X. (2024a). The mechanisms of ferroptosis and its role in atherosclerosis. Biomed. and Pharmacotherapy = Biomedecine and Pharmacotherapie 171, 116112. 10.1016/j.biopha.2023.116112 38171246

[B60] Xu M. Zhang D. Yan J. (2024b). Targeting ferroptosis using Chinese herbal compounds to treat respiratory diseases. Phytomedicine International Journal Phytotherapy Phytopharmacology 130, 155738. 10.1016/j.phymed.2024.155738 38824825

[B61] Xue M. Yang L. Kou N. Miao Y. Wang M. Zhao Q. (2015). The effect of xuefuzhuyu oral liquid on aspirin resistance and its association with rs5911, rs5787, and rs3842788 gene polymorphisms. Evidence-based complementary and alternative medicine. eCAM 2015, 507349. 10.1155/2015/507349 26495016 PMC4606155

[B62] Yang W. S. Stockwell B. R. (2016). Ferroptosis: death by lipid peroxidation. Trends Cell Biology 26 (3), 165–176. 10.1016/j.tcb.2015.10.014 26653790 PMC4764384

[B63] Yu W. Ilyas I. Hu X. Xu S. Yu H. (2022). Therapeutic potential of paeoniflorin in atherosclerosis: a cellular action and mechanism-based perspective. Front. Immunology 13, 1072007. 10.3389/fimmu.2022.1072007 PMC9811007 36618414

[B64] Yu X. Qin W. Cai H. Ren C. Huang S. Lin X. (2024). Analyzing the molecular mechanism of xuefuzhuyu decoction in the treatment of pulmonary hypertension with network pharmacology and bioinformatics and verifying molecular docking. Comput. Biology Medicine 169, 107863. 10.1016/j.compbiomed.2023.107863 38199208

[B65] Yuan R. Wang Y. Cong W. H. Chen K. J. (2017). Treatment of cardiovascular disease with xiongshao capsule. Zhongguo Zhong Yao Za Zhi = Zhongguo Zhongyao Zazhi = China Journal Chin. Materia Medica 42 (4), 640–643. 10.19540/j.cnki.cjcmm.2017.0017 28959830

[B66] Yuan R. Shi W. Xin Q. Yang B. Hoi M. P. Lee S. M. (2018). Tetramethylpyrazine and paeoniflorin inhibit oxidized LDL-induced angiogenesis in human umbilical vein endothelial cells via VEGF and notch pathways. Evidence-based Complementary Alternative Medicine eCAM. 2018, 3082507. 10.1155/2018/3082507 30584451 PMC6280302

[B67] Yuan R. Li Z. H. Huang M. W. Li P. Q. Miao Y. Mo H. (2023a). Intervention effect of chuanxiong-chishao herb pair on circRNA/lncRNA expression profile in a myocardial infarction-atherosclerosis model. Zhongguo Zhong Yao Za Zhi = Zhongguo Zhongyao Zazhi = China Journal Chin. Materia Medica 48 (14), 3890–3903. 10.19540/j.cnki.cjcmm.20230306.703 37475081

[B68] Yuan R. Xin Q. Ma X. Yu M. Miao Y. Chen K. (2023b). Identification of a novel angiogenesis signalling circSCRG1/miR-1268b/NR4A1 pathway in atherosclerosis and the regulatory effects of TMP-PF *in vitro* . Mol. Basel, Switz. 28 (3), 1271. 10.3390/molecules28031271 PMC9919304 36770940

[B40] Yuan R. Shi W. Xin Q. Weihong C. (2019). Progress on rhizoma chuanxiong-radix paeoniae rubra herb pair. Glob. Tradit. Chin. Med. 12 (05), 808–811. 10.3969/j.issn.1674-1749.2019.05.047

[B69] Yuerong J. Yuanhua X. Jingchun Z. Dazhuo S. (2015). CHEN keji's medication laws for treating blood stasis pattern of cardiovascular diseases: a data mining research. J. Traditional Chin. Med. 56 (05), 376–380. 10.13288/j.11-2166/r.2015.05.005

[B70] Zhang M. Liu J. Guo W. Liu X. Liu S. Yin H. (2016). Icariin regulates systemic iron metabolism by increasing hepatic hepcidin expression through Stat3 and Smad1/5/8 signaling. Int. Journal Molecular Medicine 37 (5), 1379–1388. 10.3892/ijmm.2016.2545 27035325

[B71] Zhang M. Sun M. Y. Guo C. Y. Wang J. S. Xu F. Q. Yin H. J. (2019). Effect of tetramethylpyrazine and hyperlipidemia on hepcidin homeostasis in mice. Int. Journal Molecular Medicine 43 (1), 501–506. 10.3892/ijmm.2018.3968 30387806

[B72] Zhang Q. Liu J. Duan H. Li R. Peng W. Wu C. (2021). Activation of Nrf2/HO-1 signaling: an important molecular mechanism of herbal medicine in the treatment of atherosclerosis via the protection of vascular endothelial cells from oxidative stress. J. Advanced Research 34, 43–63. 10.1016/j.jare.2021.06.023 PMC8655139 35024180

[B73] Zhang Y. Ma C. He L. Liao L. Guo C. Wang C. (2022). Tetramethylpyrazine protects endothelial injury and antithrombosis via antioxidant and antiapoptosis in HUVECs and zebrafish. Oxidative Medicine Cellular Longevity 2022, 2232365. 10.1155/2022/2232365 35898617 PMC9313999

[B74] Zhang J. Nie C. Zhang Y. Yang L. Du X. Liu L. (2024). Analysis of mechanism, therapeutic strategies, and potential natural compounds against atherosclerosis by targeting iron overload-induced oxidative stress. Biomed. and Pharmacotherapy = Biomedecine and Pharmacotherapie 177, 117112. 10.1016/j.biopha.2024.117112 39018869

[B75] Zhang Y. Huang R. Liu X. Cai M. Su M. Cheng Y. (2025). Taohong siwu decoction ameliorates abnormal uterine bleeding via inhibiting ACSL4-mediated ferroptosis. J. Ethnopharmacology 339, 119130. 10.1016/j.jep.2024.119130 39566864

[B76] Zheng G. H. Liu J. P. Chu J. F. Mei L. Chen H. Y. (2013). Xiongshao for restenosis after percutaneous coronary intervention in patients with coronary heart disease. Cochrane Database Systematic Reviews 2013 (5), Cd009581. 10.1002/14651858.CD009581.pub2 23728695 PMC11246721

[B77] Zhi W. Liu Y. Wang X. Zhang H. (2023). Recent advances of traditional Chinese medicine for the prevention and treatment of atherosclerosis. J. Ethnopharmacology 301, 115749. 10.1016/j.jep.2022.115749 36181983

[B78] Zhu Z. Li J. Song Z. Li T. Li Z. Gong X. (2024). Tetramethylpyrazine attenuates renal tubular epithelial cell ferroptosis in contrast-induced nephropathy by inhibiting transferrin receptor and intracellular reactive oxygen species. Clin. Science Lond. Engl. 138 (5), 235–249. 10.1042/cs20231184 PMC10899005 38357976

